# Ventricular Septation and Outflow Tract Development in Crocodilians Result in Two Aortas with Bicuspid Semilunar Valves

**DOI:** 10.3390/jcdd8100132

**Published:** 2021-10-15

**Authors:** Robert E. Poelmann, Adriana C. Gittenberger-de Groot, Charissa Goerdajal, Nimrat Grewal, Merijn A. G. De Bakker, Michael K. Richardson

**Affiliations:** 1 Sylvius Laboratory, Department of Animal Sciences and Health, Institute of Biology, University of Leiden, Sylvi-usweg 72, 2333BE Leiden, The Netherlands; charissa_go@hotmail.com (C.G.); M.A.G.de.Bakker@biology.leidenuniv.nl (M.A.G.D.B.); m.k.richardson@biology.leidenuniv.nl (M.K.R.); 2 Department of Cardiology, Leiden University Medical Center, Albinusdreef 2, P.O. Box 9600, 2300RC Leiden, The Netherlands; a.c.gittenberger-degroot@lumc.nl; 3 Department of Cardiothoracic Surgery, Leiden University Medical Center, Albinusdreef 2, P.O. Box 9600, 2300RC Leiden, The Netherlands; N.Grewal@lumc.nl

**Keywords:** endocardial cushions, semilunar valves, outflow tract, cartilage, foramen of Panizza, left aorta, right aorta, pulmonary trunk, pharyngeal arch arteries, coronary arteries

## Abstract

**Background**: The outflow tract of crocodilians resembles that of birds and mammals as ventricular septation is complete. The arterial anatomy, however, presents with a pulmonary trunk originating from the right ventricular cavum, and two aortas originating from either the right or left ventricular cavity. Mixing of blood in crocodilians cannot occur at the ventricular level as in other reptiles but instead takes place at the aortic root level by a shunt, the foramen of Panizza, the opening of which is guarded by two facing semilunar leaflets of both bicuspid aortic valves. **Methods**: Developmental stages of Alligator mississipiensis, Crocodilus niloticus and Caiman latirostris were studied histologically. **Results and Conclusions**: The outflow tract septation complex can be divided into two components. The aorto-pulmonary septum divides the pulmonary trunk from both aortas, whereas the interaortic septum divides the systemic from the visceral aorta. Neural crest cells are most likely involved in the formation of both components. Remodeling of the endocardial cushions and both septa results in the formation of bicuspid valves in all three arterial trunks. The foramen of Panizza originates intracardially as a channel in the septal endocardial cushion.

## 1. Introduction

The bicuspid aortic valve (BAV) is the most common congenital cardiac malformation in humans with a frequency of 0.5–2% [1]. Furthermore, the malformation is associated with aortic aneurysm in 60–80% of the BAV population later in life [2] and is, therefore, of clinical importance [3]. Much is known about the embryonic origin of abnormal leaflet numbers associated with genetic mutations in the human population [4], but also in genetic models of mice such as GATA5 [5], Krox20 [6], Notch [7], aggrecan [8], periostin [9] and nitric oxide synthase [10], in hamsters [11] and also in bird hemodynamics [9,12]. It is evident that several cell populations act in concert during the formation of the semilunar valves. These include the endocardial cushions, neural crest cells and second heart field. Less is known about the development of the arterial valves in reptiles where bicuspidy is the rule [13,14]. Our restricted level of knowledge is comprehensible as a lack of marker experiments is hampering cell lineage research in the reptilian setting, while genetic mutants specifically affecting outflow tract development have not been published to our knowledge. This does not mean that tools are unavailable. In an evolutionary context, the comparison of development may elucidate the characteristics of bicuspidy in different taxa. Here, we report the development of the arterial valves in three species of crocodilians (*Crocodylus niloticus, Alligator mississipiensis* and *Caiman latirostris*) to be discussed against mammalian and avian backgrounds. The remodeling of the endocardial cushions will be investigated during outflow tract septation, leading to the separation of three main arteries. These are the pulmonary trunk and left-sided aorta both emerging from the right ventricle, and the right-sided aorta emerging from the left ventricle. All three arteries contain bicuspid valve leaflets. Finally, the septal outflow tract cushion (in crocodilians only) is further specialized. Here, we find the origin of two septal components involved in the separation of the three main arterial stems and, furthermore, the intracardiac origin of a channel between the left- and right-sided circulations, i.e., both aortas. After separation and remodeling of the outflow tract, this shunt, known as the foramen of Panizza (first described by the Italian anatomist Bartolomeo Panizza, 1785–1867 [15,16]) connects the roots of the left and right aortas that, in specific circumstances, may convey blood from the right ventricle to the main circulation [17,18,19] and sometimes vice versa.

### 1.1. General Description

In crocodilians, the presence of three interconnected ventricular cava, as it is common in reptiles, is apparent in the early stages. However, during the development described here, this distinction becomes lost because interventricular septation takes place, and the resulting division is conveniently described as the left and the right ventricle. This comes with a consequence as, in crocodilians, two aortas persist (as in other reptiles), each deriving from one of those ventricles. To avoid complications related to left/right-sidedness in the body and right/left origin of the respective aortas, we have chosen to use the term “systemic aorta” (sAo) for the morphologically right-sided aorta that derives from the left ventricle, while “visceral aorta” (vAo) denominates the left-sided aorta that branches from the right ventricle (similar to Poelmann [20]. Note: Cook et al. [21] countered the sidedness problem differently by employing the terms “left-ventricular aorta” and “right-ventricular aorta”, respectively). Our choice is further substantiated by the observation that the right ventricle perfuses both the abdominal and thoracic viscera as the left-sided aorta (-> vAo) will perfuse the intestines or viscera [22] and the right-sided pulmonary blood will perfuse the lungs (note that viscera and lungs are both “endoderm-derived” organ systems), whereas the left ventricle and right-sided aortic blood (-> sAo) will mostly perfuse the combination of the body wall, extremities and head/neck region (Supplemental Figure S1, adapted from [9]) 

### 1.2. Description of Stages

**Figure 1:**
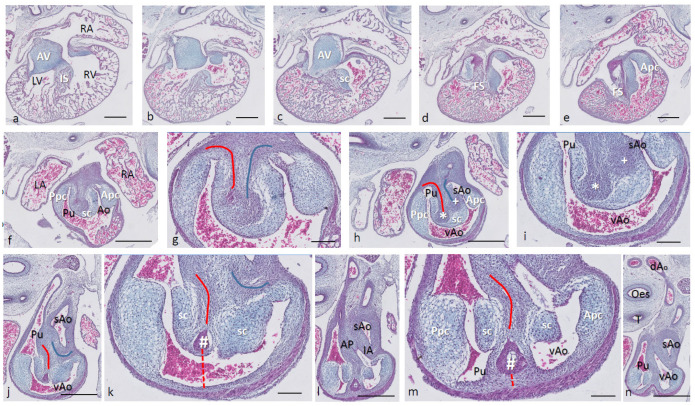
Ferguson stage 17. HE Alcian blue-stained serially sectioned embryo. Figure (**a**–**n**) from AV canal to pharyngeal arch arteries. Generally, the endocardial cushions stand out because of the Alcian blue staining. Figure (**a**), myocardium is spongious with a thin compact outer layer. The inlet septum is the most conspicuous part of the interventricular septum located between the left- and right-sided parts of the undivided common ventricle. Figure (**b**,**c**), the outflow tract septal cushion becomes visible. Figure (**d**,**e**), the folding septum only becomes clear more distally. Figure (**f**), pulmonary and aortic parietal OFT cushions become apparent, flanking the pulmonary channel and the yet common aortic channel. Figure (**g**), magnified Figure (**f**, in the large centrally located septal cushion, two streams of condensed mesenchyme, on the pulmonary side (in red) and the aortic side (in blue). The septal cushion becomes subdivided over the main arterial channels. Figure (**h**,**i**), both streams end separately in a bulbous structure as the basis for the AP septum (*) and IA septum (+). Figure (**j**), magnified in Figure (**k**), the AP stream meets the ventral myocardial spur (#). Figure (**l**), magnified in (**m**), the myocardial spur is part of the ventral myocardium indicated by the red dotted line. At this level, the AP septum is completed. Figure (**n**), pharyngeal arch arteries are separated, and the connection to the dorsal aorta is present. Key to the symbols **#** ventral myocardium, part of AP septum; * (presumably) left-sided neural crest cells, part of AP septum; **+** (presumably) right-sided neural crest, part of IA septum; **Δ** dorsal myocardium, part of AP septum. Magnification Figure (**a**–**e**,**f**,**h**,**j**,**l**,**n**), bar 500 µm; (**g**,**i**,**k**,**m**), 200 µm.

**Figure 2:**
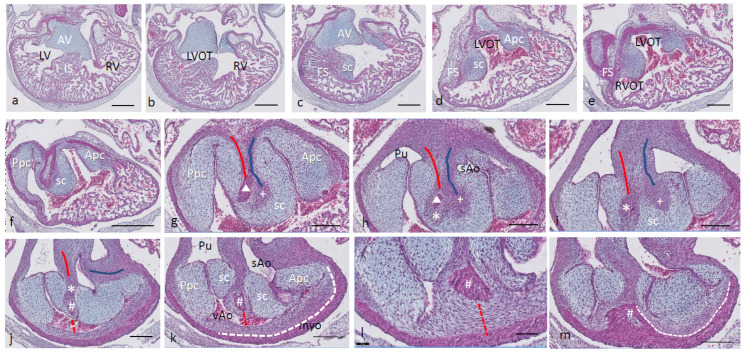
Ferguson stage 19. HE Alcian blue-stained serially sectioned embryo. Figure (**a**–**m**) from AV canal to OFT. Figure (**a**), ventricular myocardial inlet septum attached to the central AV cushions between the left and right ventricles. The myocardium is spongy but for the thin outer compact layer. Figure (**b**), more ventrally, the interventricular communication is visible, while the left ventricular outflow tract becomes apparent. Figure (**c**), muscular folding septum appears as continuation of the inlet septum. The septal OFT cushion is located at the tip of the folding septum. Figure (**d**), septal cushion with Alcian blue-stained condensed mesenchyme. The aortic parietal cushion appears on the other side of the LVOT. Figure (**e**), the septal cushion is located between both outflow tracts, while the folding septum has shifted to the right side. Figure (**f**), in the RVOT the pulmonary parietal cushion is present, and the folding septum including the septal cushion takes up a dorsal position. Figure (**g**), the most cranial remnant of the muscular folding septum is indicated (Δ). The adjacent condensed mesenchyme (+) belonging to the interaortic septum has appeared in the septal cushion. Figure (**h**), a second element of condensed mesenchyme (*) appears ventral to the disappearing myocardium (Δ). Figure (**i**), two streams of condensed mesenchyme as part of the aorto-pulmonary septum (*) and the interaortic septum (+) separate the septal cushion into three subcushions. Figure (**j**), more distally, only the AP septum continues and receives the myocardium (#) from the ventral wall indicated by the red dotted line. Figure (**k**), the condensed mesenchyme of the AP septum is not present anymore, but the myocardial component has enlarged (#). The pulmonary trunk, the visceral aorta and the systemic aorta are indicated, as are the endocardial cushion components. Only the visceral aorta is still encased in the myocardium, but note that the arterial wall of the visceral aorta continues within the myocardial tube (dashed line). Figure (**l**), the visceral and systemic aortas are separated at this level by the AP septum (red line). Figure (**m**), here, the AP septum is myocardialized (#). Key to the symbols **#** ventral myocardium, part of AP septum; * (presumably) left-sided neural crest cells, part of AP septum; + (presumably) right-sided neural crest, part of IA septum; Δ dorsal myocardium, part of AP septum. Magnification Figure (**a**–**f**), bar 500 µm; (**g**–**m**), bar 200 µm; (**g**–**m**), 200 µm; (**l**), 100 µm.

**Figure 3:**
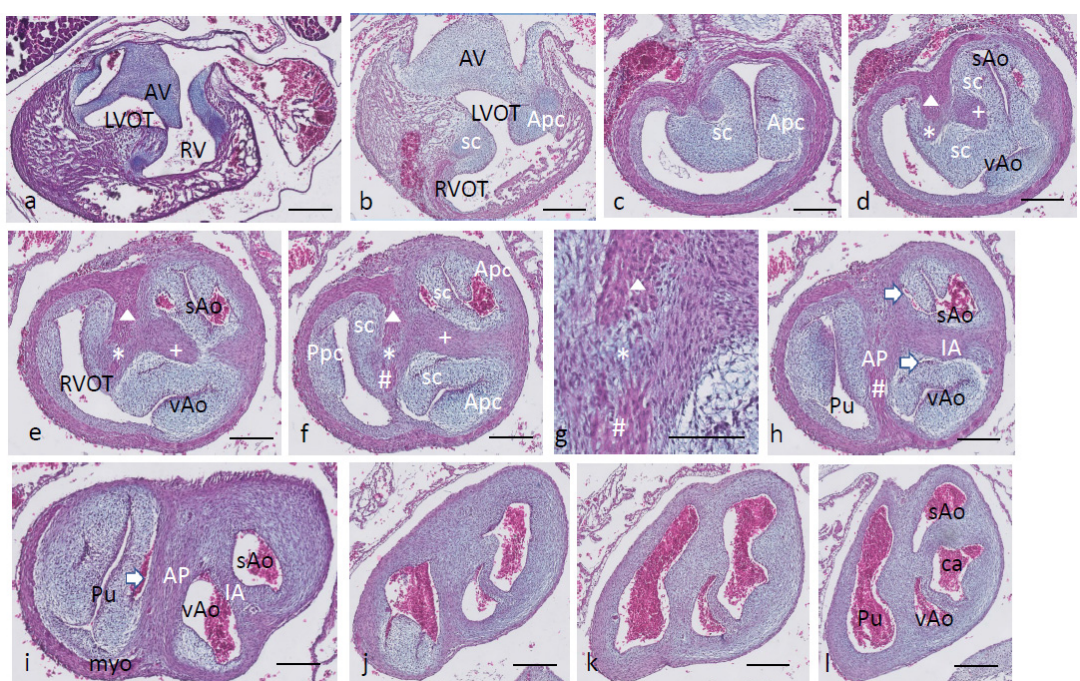
Ferguson stage 20, Figure (**a**–**l**) from AV canal to OFT. Figure (**a**), both in the septal and aortic parietal cushions, cartilage is forming (dark blue) which is less evident more distally (Figure (**b**)). Figure (**c**), the septal cushion containing condensed mesenchyme has a central position. Figure (**d**,**e**), the septal cushion becomes subdivided by both prongs of condensed mesenchyme, belonging to the IA septum (+) and AP septum (*), where the remnant of the folding septum is indicated (Δ). Figure (**f**), the condensed mesenchyme of the IA septum bridges the gap to the aortic parietal cushion whereby the systemic and visceral aortas become separated at this level. The AP septum now contains 3 elements. Enlargement in Figure (**g**), condensed mesenchyme (*), flanked by the myocardium of the folding septum (Δ) and the ventral myocardium (#). Figure (**h**), both IA and AP septa are fully developed at this level. The arrows indicate the sinus of Valsalva in the systemic and visceral aortas. Figure (**i**), the arrow indicates a sinus of Valsalva in the pulmonary trunk. Figure (**j**–**l**) illustrate the branching of the arterial tree, including the pulmonary arteries, and the carotid arteries from the systemic aorta. The visceral aorta does not branch further. Key to the symbols **#** ventral myocardium, part of AP septum; * (presumably) left-sided neural crest cells, part of AP septum; **+** (presumably) right-sided neural crest, part of IA septum; Δ dorsal myocardium, part of AP septum. Magnification Figure (**a**–**f**,**h**–**l**), 500 µm; (**g**), 100 µm.

**Figure 4:**
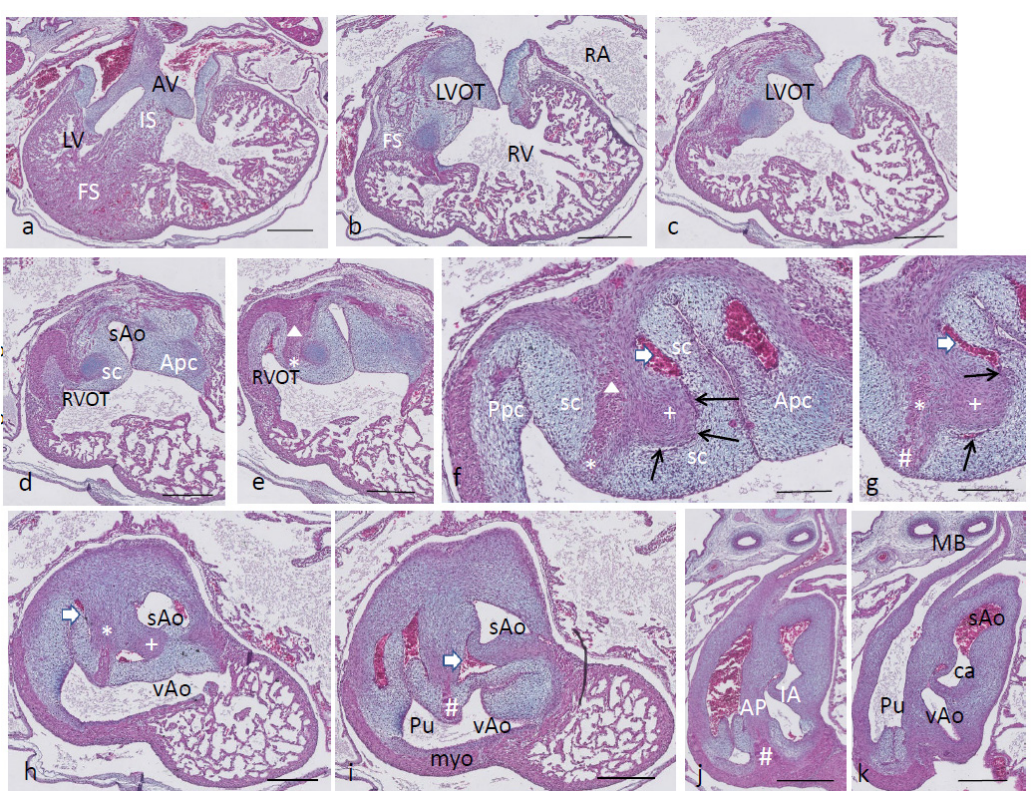
Ferguson stage 21, from AV canal to the arterial tree. Figure (**a**), the ventricular septum shows its two parts: the inlet septum attached to the central AV cushions and the more ventro-distally located folding septum. Figure (**b**,**c**), the septal cushion already showing cartilage differentiation (dark blue) located on the tip of the folding septum. Figure (**d**,**e**), the right ventricular outflow tract rotates to the right, displacing the folding septum (Δ) to the dorsal wall. Figure (**f**,**g**), the AP (*) and IA (+) streams of condensed mesenchyme are visible. The sinus of Valsalva (white arrow) and the tunnel of the foramen of Panizza (black arrows) are indicated. Figure (**h**–**j**), more distally, the AP and IA septa become completed by fusion of the septal cushion with the parietal cushion. Figure (**k**), branching of the arterial tree. Key to the symbols **#** ventral myocardium, part of AP septum; * (presumably) left-sided neural crest cells, part of AP septum; **+** (presumably) right-sided neural crest, part of IA septum; Δ dorsal myocardium, part of AP septum. Magnification Figure (**a**–**e**,**h**–**k**), bar 500 µm; (**f**,**g**), bar 200 µm.

**Figure 5:**
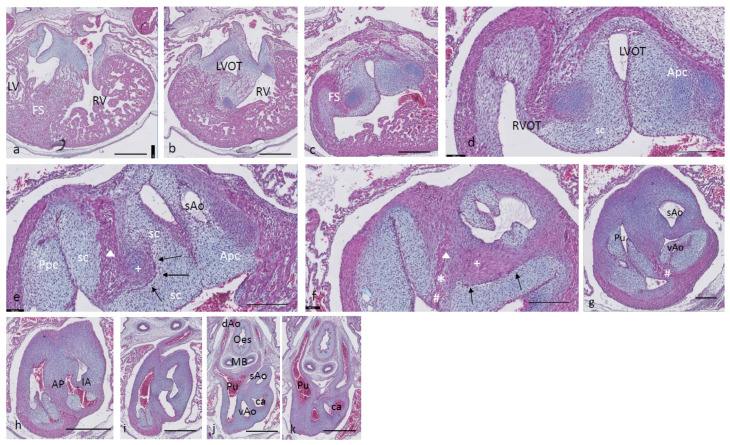
Ferguson stage 22, Figure (**a**–**i**), from AV canals to the arterial tree. Figure (**a**,**b**), septal cushion with cartilage between left and right ventricles. Figure (**c**), cartilage differentiation in both the septal and aortic parietal cushions. Figure (**d**), enlargement of Figure c, the septal cartilage related to the IA septum is located on the left aortic side of the folding septum. Figure (**e**,**f**), the AP (*), (Δ), (#) and the IA (+) septa are completely visible, containing their various elements. The tunnel of the foramen of Panizza is present (black arrows). Figure (**g**–**k**) demonstrate the branching pattern of the arterial tree. Key to the symbols **#** ventral myocardium, part of AP septum; * (presumably) left-sided neural crest cells, part of AP septum; + (presumably) right-sided neural crest, part of IA septum; Δ dorsal myocardium, part of AP septum. Magnification Figure (**a**–**c**,**h**–**k**), bar 500 µm; (**d**–**g**), 200 µm.

**Figure 6:**
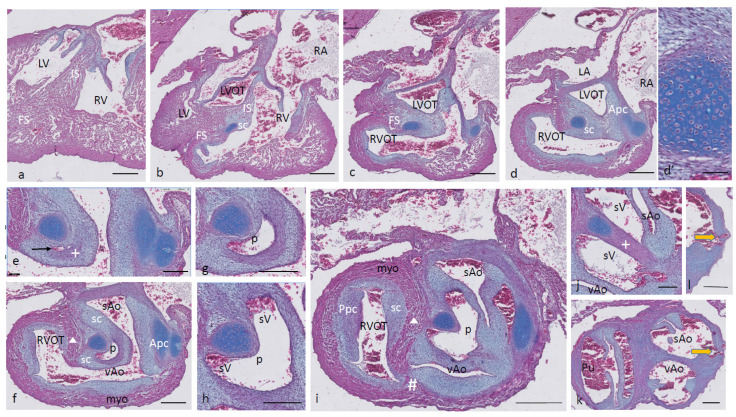
Ferguson stage 24, Figure (**a**–**l**), from AV valves to semilunar valve level. Figure (**a**,**b**), the AV cushions have elongated to form free-edged valve leaflets. Figure (**c**,**d**), folding septum including septal cushion is centrally located between left and right ventricular compartments. Cartilage-rich prongs in both OFT cushions. Note that the parietal cushion now contains two cartilaginous centers. Figure (**d’**) shows a higher magnification of the hypertrophic chondrocytes. Figure (**e**–**h**), the tunnel of the foramen of Panizza (p) is shown rounding the septal cartilage and joining the sinus of Valsalva (sV) of both aortas. Note that the cartilage is surrounded by a fibrous capsule. Figure **i**, the AP septum is completed. Figure (**j**), the IA septum is completed. Figure (**k**,**l**), coronary ostia (yellow arrows) are found in the parietal wall of the systemic aorta, but not the visceral aorta. Key to the symbols **#** ventral myocardium, part of AP septum; (presumably) left-sided neural crest cells, part of AP septum; **+** (presumably) right-sided neural crest, part of IA septum; Δ dorsal myocardium, part of AP septum. Magnification Figure (**a**), bar 1mm; (**b**–**d**,**i**), bar 500 µm; (**e**–**h**,**j**–**l**), bar 200 µm; (**d’**), bar 50 µm.

**Figure 7:**
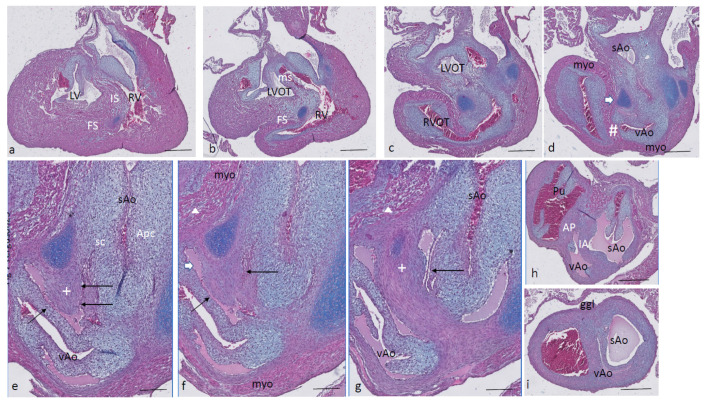
Ferguson stage 25, Figure (**a**–**i**), from AV to semilunar valve leaflets. The myocardium of this specimen is more compact compared to the earlier stages described here. Figure (**a**,**b**), the left and right ventricular compartments are completely septated by the combination of the folding septum, inlet septum and mesenchymal septum. Figure (**c**,**d**), the left and right ventricular outflow tracts and the ensuing arterial trunks are separated by the AP septum and the IA septum. The aortic parietal cushion contains two cartilaginous elements. Figure (**e**,**f**) show the narrow tunnel of the foramen of Panizza (arrows) through the IA septum (+). Figure (**g**), the IA septum at this level is uninterrupted, with the flanking sinus of Valsalva of the visceral and systemic aortas. Figure (**h**,**i**), both AP and IA septa are obvious, and the lumen of the visceral aorta is very narrow in this specimen. Key to the symbols **#** ventral myocardium, part of AP septum; (presumably) left-sided neural crest cells, part of AP septum; **+** (presumably) right-sided neural crest, part of IA septum; **Δ** dorsal myocardium, part of AP septum. Magnification Figure (**a**–**d**,**h**,**i**), bar 500 µm; (**e**–**g**), bar 200 µm.

**Figure 8:**
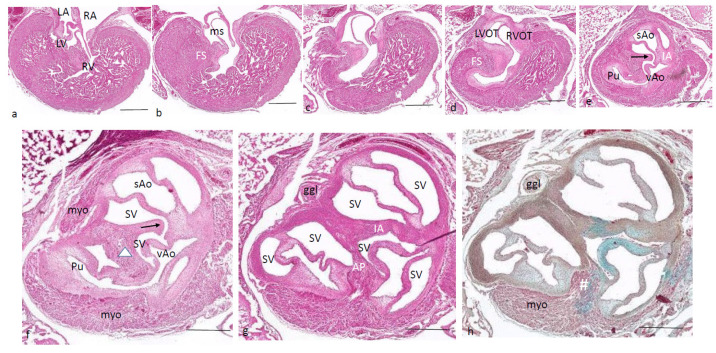
Alligator embryo older > than F stage 25. HE-stained Figure (**h**) is, in addition, Alcian blue stained. Figure (**a**–**h**) from AV valves to semilunar valves. Figure (**a**–**d**), the mesenchymal septum is inserted between the right and left ventricular compartments separating both ventricles. It reaches from the tip of the folding septum near the septal cushion (in Figure (**a**)) towards the IA septum (in Figure (**d**,**e**)). The communication between visceral and systemic aortas is still patent (Figure (**d**)). Figure (**e**,**f**), here, the IA septum is interrupted by the tunnel of the foramen of Panizza (arrow). Figure (**g**,**h**), both the AP septum and the IA septum are complete at this level. Key to the symbols **#** ventral myocardium, part of AP septum; (presumably) left-sided neural crest cells, part of AP septum; **+** (presumably) right-sided neural crest, part of IA septum; **Δ** dorsal myocardium, part of AP septum. Magnification Figure (**a**–**e**), bar 200 µm; (**f**–**h**), bar 500 µm.

The descriptions are illustrated from caiman embryos (Figure 1, Figure 2, Figure 3, Figure 4, Figure 5, Figure 6 and Figure 7); only Figure 8 is from an alligator. We chose to start our study with stage 17, although several elements (endocardial cushions, parts of the muscular interventricular septum) already appear earlier. In St 17, the elements start to make up a comprehensible combination leading to complete septation.

Ferguson stage 17. The right and left ventricles are dorsally intersected by the inlet septum (Figure 1a), but otherwise not separated, as the interventricular communication presents itself clearly (Figure 1b). The ventricular inlet septum is fused with the large central AV cushion complex, while the folding septum [20] can hardly be discerned (Figure 1a–c). We have defined the folding septum [20] as a part of the muscular interventricular septum particularly located between the right and left ventricular outflow tracts. Slightly more downstream, the septal outflow tract cushion (sc) becomes apparent, together with the flanking aortic and pulmonary parietal outflow tract cushions (Apc and Ppc) (Figure 1d–f). In the septal cushion, two streaks of condensed mesenchymal cells are evident, one on the pulmonary side (red curve in Figure 1g–i), and the other on the aortic side (blue curve). At their tips (* and + in Figure 1h,i), both contain a histologically very dense cluster of cells. In a recent study [20], we showed the presence of neural crest cells in this dense cluster by the expression of AP2alpha, a known marker of NC. The pulmonary streak or aorto-pulmonary (AP) septum might contain left-sided NC-derived cells, while the aortic streak or interaortic (IA) septum probably contains right-sided NC cells, as it will be discussed later. The curved lumen of the outflow tract (Figure 1h,i) can be divided into the pulmonary outflow tract (from the right ventricle), the visceral aorta (vAo, also from the right ventricle) and the systemic aorta (sAo, left ventricle). Separation of the outflow tracts (Figure 1j) takes place by 1. the AP septum including a histologically distinguishable ventral myocardial spur (# in Figure 1j–n) located between the pulmonary trunk and the aortas, and 2. the IA septum between sAo and vAo. In the thorax, no further branches will be found emanating from the vAo. The sAo, however, branches further in both carotid arteries (not depicted here).

Ferguson stage 19. The interventricular situation (Figure 2a,b) has not advanced very much compared to stage 17 as the interventricular communication is still open and the folding septum (FS) remains inconspicuous (Figure 2c). The septal outflow tract cushion (sc) acquires a dense core (Figure 2c) preceding the differentiation of cartilage in the next stage. The interconnected lumina of the LVOT and RVOT are easily recognized (Figure 2d,e), interrupted by the large septal cushion (sc). The root of the Pu seems to be hconnected sideways (Figure 2d–f) to the ventricular segment as a result of folding of this part of the interventricular septum. The septal cushion is located at the top of the folding septum (Figure 2c,d) that becomes more clear in the next stage. In the as yet common outflow tract, the three arteries become separated by the AP septum and the IA septum. In the AP septum, the region of the originally dense cluster of cells (see Figure 2h *) surrounds a dorsal spur of the myocardium (Δ in Figure 2g,h), being the cranial tip of the folding septum. This dorsal myocardial spur has not yet fused with the ventral myocardial spur (# Figure 2j–m and dashed red line (Figure l) to show the continuation with the ventral wall) as there is a “window”, free from the myocardium in between (* in Figure 2h–j). AP separation and IA separation are seemingly spatially independent of each other (Figure 2i,j). The septal cushion is distally completely bisected by the AP septum (Figure 2k). The right-sided IA septum deviates between both aortas with a dense cell cluster at its tip (+ in Figure 2h,i).

The transition of the cardiac tube to the arterial vascular wall deserves special attention. It is evident that the arterial wall extends inside the cardiac tube over a considerable distance estimated to be about 200 µm on the pulmonary side and even about 400 µm on the aortic side (white dashed line in Figure 2k). Downstream, this appearance is even more substantiated (Figure 2m), where nearly the complete ventral wall of the vAo seems to consist of a double layer of the vessel wall and myocardium (white dashed line) with hardly a thin endocardial cushion tissue. This continues to an estimated depth of 350–400 µm into the myocardial tube. As a consequence, we are dealing with two concentric tubes, a myocardial circumference surrounding an arterial wall.

Ferguson stage 20. The interventricular communication between the LVOT and RV is almost closed (Figure 3a). The dense core in the septal and in the aortic parietal cushions starts to differentiate into cartilage, as shown by intense Alcian blue staining (Figure 3a–c), although more downstream, this differentiation is not apparent yet (Figure 3d,e). Here, the IA septum consists of densely packed right-sided neural crest cells, as described in the earlier stages (+ in Figure 3d). The AP septum now contains a clear core of the myocardium (Δ in Figure 3d–f) as part of the incurving dorsal wall of the myocardial tube. Where the septal cushion has connected with the ventral wall, the ventral myocardial spur (# in Figure 3f–h) is also present. The two spurs do not contact each other (* depicts the mesenchymal “window” between the myocardial spurs indicated by Δ and # in Figure 3g). The AP and IA septa deviate (Figure 3h,i) to continue outside the heart as the mesenchymal vessel walls of the respective arteries (Figure 3j–l). The common stem of the carotid arteries (ca) branches from the systemic aorta (Figure 3l). Two sinuses of Valsalva (white arrow) are present in the OFT cushions of the systemic aorta and the pulmonary trunk (Figure 3h,i) as the first sign of the forming semilunar valve leaflets. The situation in the visceral aorta is slightly different as in the parietal cushion, a sinus of Valsalva is still lacking, but in the septal cushion, a very narrow slit is seen, representing the sinus of Valsalva that is connected to the main lumen of the vAo, probably being the first sign of the origin of the foramen of Panizza (see description in the next stage, stage 21).

Ferguson stage 21. The ventricular inflow septum (IS) is seen as the continuation of the folding septum (Figure 4a,b), beyond the topping cartilage of the septal cushion (Figure 4b–e). Another smaller piece of cartilage is found in the aortic parietal cushion (Figure 4c,d). In the outflow tract, the pulmonary side becomes separated from the aortic side by the large septal cushion, containing the elements of the AP septum (* and Δ in Figure 4e–h) as well as the IA septum (+ in Figure 4f–h). Here, an interesting feature becomes apparent, known as the foramen of Panizza (FOP, black arrows in Figure 4f,g), connecting the two septal sinuses of Valsalva of the sAo (white arrow) and vAo. Actually, the FOP in this stadium is a very narrow channel, about 100 µm high in the cranio-caudal direction and about 200 µm between both sinuses. The channel surrounds the advancing tip of the IA (+ in Figure 4f,g) that, at this level, is completed in the arterial direction only (Figure 4h,i). Further downstream, the myocardial tube is replaced by the arterial vessel walls in the concentric manner described above. The stem of the carotid arteries branches from the sAo (Figure 4j,k). 

Ferguson stage 22. Most of the features described for stage 21 apply to this stage. The folding septum is more advanced (Figure 5a). The condensed mesenchyme in the septal cushion is further differentiated into cartilage (Figure 5b–d), even reaching the level of the FOP (Figure 5d,e, black arrows). The FOP is seen as a very narrow channel rounding the cartilaginous tip at the base of the IA (Figure 5d,e). The condensed mesenchyme in the aortic parietal cushion is advanced and now contains two cartilaginous centers (Figure 5c), one in the continuation of the aortic vessel wall, and the second one in the cushion mesenchyme. The ventral and dorsal myocardial spurs (# and Δ in Figure 5e) have joined with no mesenchymal window in between anymore, implying that a small myocardial bridge has been established in the AP septum. The IA septum does not acquire a myocardial component in contrast to the AP septum (Figure 5e–g). The branching of the main arterial stems is demonstrated in Figure 5h–i. The pulmonary trunk is divided into the sixth left and right pulmonary arch arteries (PAA6, Figure 5h), and the vAo, being the left fourth PAA, does not branch further, whereas the stem of the carotid arch arteries splits from the right fourth PAA, being the sAo (Figure 5h–i). 

Ferguson stage 24. The folding and inlet septa meet each other at an angle with the septal outflow tract cushion as a hinge (Figure 6a,b). The inlet septum continues between the LVOT and the right ventricle (Figure 6b,c), while the septal and aortic parietal cushions have fused over a short distance (Figure 6d) to reopen again further downstream. A mesenchymal interventricular septum is not apparent yet, in contrast to stage 25. The IA septum is likewise not fully developed, leaving a lumen contact between the sAo and vAo (Figure 6e,f). Both imply that left–right separation has not been established yet. The cartilage prongs are well differentiated both in the septal cushion and aortic parietal cushion, and in the latter, two cartilage elements are present (Figure 6e,f), as in earlier stages. The FOP in the septal cushion is wide open, connecting both the vAo’s and sAo’s sinus of Valsalva (Figure 6f–i). Further downstream, the septal cartilage partly penetrates the interaortic septum (Figure 6j). In the root of the sAo, the double orifice of the coronary artery is present (orange arrow in Figure 6k,l), splitting immediately in a descendent and a circumflex branch (Figure 6l).

Ferguson stage 25. The spongious myocardia of the left and right ventricles have increased considerably in mass (Figure 7a,b). The cartilage of the septal outflow tract cushion is embedded deep apically between the left and right ventricles squeezed between the inlet and folding segments (Figure 7a). The presence of the mesenchymal septum (even more visible in the next stage) between the left and right ventricles shows that complete septation has taken place. Even in this stage, the complete separation of the sAo and vAo by the interaortic septum has not been established as there is a narrow lumen contact seen between the LVOT and RVOT (Figure 7b–h) and, as a consequence, also between the (left ventricular) sAo and (right ventricular) vAo. This implies a complete interventricular septation from now on, but for the narrow connection between the sAo and vAo provided by the FOP (black arrows in Figure 7e–g) and the narrow lumen contact between both aortas. Functionally, in this embryo, the FOP is closed as no red blood cells are found trapped in its lumen. The vAo appears very compressed in this particular embryo (Figure 7i).

Beyond Ferguson stage 25 (alligator). In this HE-stained series, the LV and RV are completely septated, and the hallmark of this process is the mesenchymal septum extending over a considerable distance (Figure 8a–d), reaching as far as the IA septum (Figure 8d,e). In the outflow tract, the septal cushion is penetrated by the FOP (black arrow), connecting both septal sinuses of Valsalva of the sAo and the vAo (Figure 8e,f) at the level where the arterial wall is inserted into the myocardium. Slightly more downstream, the IA septum separates the two aortas, whereas the AP septum completely separates the pulmonary trunk from the vAo (Figure 8g,h). Note that Figure 8h is Alcian blue stained to demonstrate the myocardium versus the mesenchymal wall of the arteries.

## 2. Material and Methods

The *Caiman latirostris* eggs were donated by René Hedegaard, Krokodille Zoo, Denmark. These were incubated until the desired stage, fixed in formalin at 4 °C overnight, dehydrated through a graded methanol series and stored in 100% methanol at −20 °C. The *Alligator mississipiensis* embryos were obtained through a project by Mark Ferguson, deposited in the Manchester Museum, from the University of Manchester (United Kingdom) and were allowed to be used for scientific purposes. These were fixed and stored in formalin. Additional *Crocodilus niloticus* eggs were obtained from La Ferme au Crocodile, Pierrelatte (France), incubated until the desired stage and treated the same as the caiman embryos above. The total numbers of embryos studied were 8 caiman embryos, 3 crocodile embryos and 10 alligator embryos (Table 1), but in addition, many younger crocodiles were available to us. 

Only the caiman material provided a continuous series of stages, while the series of crocodile and alligator material showed gaps, allowing a useful but limited comparison of the species. This holds particularly for the immunostainings for AP2alpha (neural crest) and troponin (myocardium) published before [20]. 

Staging of the embryos of these species followed the criteria of M. Ferguson [21]. Note that the comparison with chick (Hamburger/Hamilton stage) and mouse (Theiler stage) is very coarse as the differing characteristics are mainly based on unalike external features, such as legs/wings, eye lids, feather buds or whiskers.

The thorax containing the heart and arterial trunks was excised and routinely dehydrated through ethanol. The tissue was embedded standardly in paraffin, sectioned in 7 µm serial sections, collected on objective slides and stained with hematoxylin-eosin and Alcian blue as published [20]. Finally, they were coverslipped using Eukit.

Stained sections were scanned at 40 × and made electronically available with the Philips IMS system through the Dept of Pathology, LUMC Leiden, maintained by Dr. J Oosting and B. van den Akker.

## 3. Discussion

### 3.1. The Endocardial Cushions 

More than a century ago, Hochstetter [13] provided a description of crocodilian heart development. He numbered four outflow tract endocardial cushions, Hochstetter’s #1 being the septal cushion as described here. Cushion # 2, 3 and 4 appeared as separate cushions in each of the three main arteries, which we took together as the parietal cushion, as early in development, these combine as one cushion. Only during separation does this cushion become divided over the sAo, the vAo and the pulmonary trunk. Furthermore, Hochstetter already discerned the “septum aortico pulmonale” and the “septum aorticum”. In his text, Figures 10 and 13, in Crocodilus madasgacariensis (first described in [23] which is probably an eastern divergent from C. niloticus [24]), he describes the separation of both aortas to occur before that of the AP septum. Even in our youngest stage, the AP and IA septa are found in the same embryo, albeit at different levels. Apparently, the exact timing of these events is not strictly orchestrated. This heterochrony may indicate that the different cellular players are relatively independent of each other in the formation of the individual septum components. 

### 3.2. Outflow Tract Separation

We decided that early in development, two OFT cushion complexes could be discerned, the dorsal-most septal cushion and the more ventrally located parietal cushion. The single septal cushion (Sc) hugs the folding septum; the latter might also be called the vertical or muscular septum [13,25] and is positioned between the left and right outflow tracts. The OFT cushions start with endocardial–mesenchymal transition of the lining endocardium but become subsequently invaded by “condensed mesenchyme”, as shown in this study and also in birds [26], in human [27] and in turtles [20]. The cellular origin in chicken and mouse is established to derive from the cardiac neural crest, as demonstrated in various marker studies [28,29]. The mesenchyme of the two main semilunar valve leaflets derives likewise from the cardiac neural crest, while the second heart field, important for development of the right side of the heart including the pulmonary trunk [29,30], participates in the development of the third so-called non-facing semilunar valve leaflet, as shown by the expression of Cardiac Troponin T2 [31,32] or NKx2.5 [33]. Furthermore, a part of the septation complex does not derive from the cardiac crest [34]. This is demonstrated in quail–chicken chimeras [26] in which a narrow zone of compact chick mesenchyme remains present in an otherwise quail-dominated septation complex. This narrow strip is also recognized between the two advancing myocardial components in the conal septum of our caiman AP septum (depicted by * in Figure 1i, Figure 2h, and Figure 3f, etc.). 

The neural crest cells migrate into the septal cushion from cranial and dorsal positions [10,33,35,36,37], meeting the most cranial extension of the folding septum. Sumida [38] investigated the contribution of the left- and right-sided neural crest separately by transplanting a quail crest isotopically and unilaterally into a chicken host. They observed only an ipsilateral NC contribution to the pharyngeal arch arteries and to the OFT endocardial cushions, with limited crossing to the contralateral side of the AP septum. The columns of condensed mesenchyme in the OFT cushions were derived from both sides of the NC in a complementary fashion, whereas the septal cartilage presented only right-sided NC cells. Likewise, left- and right-sided columns of NC cells converging on the OFT septal complex are observed in mouse [10] after immunostaining for AP2α, a marker of neural crest cells that we employed in crocodile and turtle embryos [20], proving that also in reptiles, NC cells migrate into the AP septal complex. These studies demonstrate the existence of separate streams of NC cells, and here, we show that these streams do not mix (red and blue in Figure 1 and Figure 2g–j). The left-sided stream is at the base of the AP septum, and the right-sided stream forms the IA septum. 

This process is correlated with the persistence of two aortas in reptiles. In birds, only the right aortic arch artery persists after pharyngeal arch remodeling, whereas in mammals, the left one persists, and the right one is further downstream, incorporated into the subclavian artery. The left stream of condensed mesenchyme stays in close proximity to the folding septum and grows out to form part of the AP septum. The right stream deviates at almost 90 degrees to initiate the IA septum. The septal cushion becomes divided into three entities, each dedicated to one of the three main arterial trunks as a result from ingrowth of the two streams of condensed mesenchyme. Lineage tracing experiments in mouse and chicken have established that in these species, the single AP septal complex derives particularly from the cardiac neural crest. In the next chapter, we argue that the participation of second heart field cells in septation is restricted.

In older stages, the parietal cushions present with two long legs, the aortic (Apc) and pulmonary parietal cushion (Ppc) along the right and the left ventricular OFT. Only upon aortic separation (in Ferguson stage 20 and beyond) does the Apc divide into two bulges dedicated to one of the aortic channels each. It is worth mentioning that the pulmonary parietal cushion is not involved in the separation process, but only in the formation of a semilunar valve leaflet.

### 3.3. Cartilage in the OFT Cushions

A prominent developmental step in crocodiles starting in the most proximal tip of the condensed mesenchyme is the differentiation into cartilage. This is already noted deep in the ventricle in Ferguson stage 20 and occurs in both the Sc and the Apc. It increases in length and diameter and becomes a dominant feature in the distal OFT. At the base of the Sc close to the folding septum, the differentiation process of cartilage increases dramatically. The non-cartilaginous condensed mesenchyme of the IA inside the Sc protrudes towards the Apc, eventually establishing contact of the Sc with the Apc. The Apc in stage 22 and more pronouncedly differentiated in stage 24 even carries a double center of cartilage. At this point, we need to address the question about the origin of the IA and the cartilage prongs. As described above, a bona fide lineage marker is not available; therefore, we have to rely on circumstantial evidence to discuss the participation of neural crest and second heart field-derived cells. In various chicken [26,28,38] and mouse [10,39] models, the contribution of neural crest cells to the OFT septal complex has been proven unequivocally. In neural crest quail–chicken chimera, cartilage was a product of transplanted quail cells differentiating at the time of hatching [38]. Neural crest cells occupy the inner media of the aorta in an adult Wnt1-Cre mouse model, and these derive from the embryonic complete media. Taken together, we postulate that the IA derives from the cardiac NC. A consequence is that the wall of the systemic aorta, being continuous with the IA, originates likewise from the NC [30,40]. The origin of the wall of the visceral aorta is not completely deducted by this reasoning. It can be argued that in the side facing the systemic aorta, NC-derived cells are also present as both aortas share the IA septum. The opposite side of the visceral aorta, however, is shared with the pulmonary trunk with the joining AP septum in between. In mice [30,34,41,42], it is known that the root of the pulmonary trunk is of mixed (NCC and SHF) origin. The OFT septal complex in mouse (Peterson 2018) and chicken [26,28,35,43], which we consider here as a fusion product of the crocodilian IA and AP septa, derives mainly from the cardiac NC. At this point, it is important to evaluate, again, the paper of Sumida [37] in which quail–chicken chimeras were made of the right or left half of the neural crest. These authors showed that a right and a left NC stream converged upon the chicken OFT septal complex but remained separate in the condensed mesenchyme. In particular, in our caiman material, two streams of condensed mesenchyme (Figure 2g–j) are present, each addressing only one of the septa involved. Furthermore, the stream in blue entered both the septal and parietal cushions [37] and is, therefore, responsible for cartilage formation in both cushions. In an earlier paper [20], we showed that the aortic and pulmonary flow dividers contain NC and second heart field cells in varying degrees and assumed that the IA septum might originate from the second heart field, and the AP septum from the neural crest. With the currently available material, we propose that the core of both septal components derives from the cardiac neural crest.

Cardiac cartilage is encountered in chicken [37] and in specific groups of mammals such as hamsters [44]. In adult otters [45] and various ungulates such as buffalo [46], white rhinoceros [47] and sheep [48], a “heart bone” is present in the fibrous trigone between left AV canal and aorta. A subgroup of diseased chimpanzees with myocardial fibrosis presented with trabecular bone or hyaline cartilage in the right fibrous trigone [49]. In (sub)adult alligators, a cartilage crown is present, nearly completely encircling the roots of both aortas [50,51], but penetrating not as deep into the ventricle as described by us. In our embryonic crocodiles, alligators and caimans, the cartilage elements have not fused, which was also the case in one juvenile crocodile of approximately 60 cm overall length. Other reptiles [52], terrapin [53,54] and turtle [20] present with cartilage prongs. We hypothesize that the elements, being aorta-bound, most likely develop from the left NC stream. Evidently, the NC condensed mesenchyme and ensuing cartilage are encased by fibroblasts (Figure 6), and the origin of these fibroblasts needs further investigation. To our knowledge, second heart field-derived cells do not have the capacity to differentiate into cartilage and neither do cultured pericardial cells [55]. In addition, fibroblasts or smooth muscle cells are found inside the myocardial OFT tube as an extension of the arterial walls (see Figure 3k, dashed line), the origin of which is also not clarified. We suggested earlier [20] that the aortic and pulmonary flow dividers, in which second heart field-derived cells also play a role [37], might be involved. Without proper markers, however, the participation of NCC and SHF-derived cells (see Figure 1g,h) remains an educated guess even when more or less related species are considered for comparison. With respect to the other potentially involved cell populations such as the second heart field and epicardial/pericardial cells, further research is warranted.

### 3.4. Bicuspid Semilunar Valves

In mammals and birds, tricuspid semilunar valves are the rule, while in reptiles, bicuspid valves are consistently present. Mammalian bicuspidy is considered a congenital malformation [32] that is often associated with a fragile wall of the ascending aorta later in life [56]. Therefore, we need to find an explanation for the bicuspid valve in our crocodilians associated with a healthy aortic wall. In development, the emerging OFT cushions present as the septal and parietal cushions. During development, these reach from a deep intraventricular position adjacent to the atrioventricular cushions distally towards the arterial pole. At the arterial pole, the septal cushion, harboring the anlage of the IA and the AP septum, becomes divided into three subcushions protruding in each arterial stem, being the pulmonary trunk, the visceral aorta and the systemic aorta. The outgrowing interaortic septum eventually meets the aortic parietal cushion, separating this cushion into two subcushions. The pulmonary parietal cushion develops singly as it is not continuous anymore with the Apc. Finally, every artery contains two cushions, one derived from the septal cushion and one from the subdivided parietal cushion. Specifically, in the visceral aorta, a small Ap cushion remnant remains after the fusion process that could be compared to an intercalated cushion. However, this does not develop into a semilunar valve leaflet, probably because this remnant will not be filled by second heart field-derived cells as is normally the case in, e.g., mice [10]. We know from chicken and mouse studies that the two main cushions are filled by mesenchymal cells derived both from epithelium–mesenchymal transition from the endocardium, and later arriving neural crest cells. The third leaflet has a second heart field-related etiology, as demonstrated in a mouse NOS3-/-model for bicuspidy [10]. In view of the human fragile aortic wall, it is probably this third/non-coronary valve leaflet’s etiology that associates with the differentiation of the outer media of the aortic wall, and that becomes prone to aneurysm formation. The inner media of the wall of the ascending aorta is NC-derived [40,57], whereas the outer media/adventita is SHF-derived [5,34]. We postulate that anomalous SHF differentiation may affect both this semilunar valve leaflet and the integrity of the ascending aorta.

### 3.5. Foramen of Panizza

Unique to crocodilian hearts is the foramen of Panizza [20,50,51,58]. This is a tunnel inside the septal cushion that penetrates the IA septum, providing a seemingly intracardiac shunt between the right and left circulations. Here, it is assumed that the matrix of the OFT endocardial cushions is produced by cardiomyocytes, similar to the origin of the AV cushions [59,60]. This does not mean that the whole OFT cushion remains within the myocardial border. The proximal part stays intramyocardially, while the distal part extends, without doubt, into the arterial tube at the level of the semilunar valve leaflets. Consequentially, the fully grown location of the FOP, being enclosed in the distal part of the septal cushion, is located outside the heart, connecting the facing sinus of Valsalva between the roots of both aortas. This has implications for the way we envisage semilunar valve development from the “massive” OFT cushion towards the “hollow” valve leaflets containing the sinus of Valsalva [32]. The proximal part of the cushions remains attached to the myocardial border, while the developing leaflets and commissures develop from the distal part of the cushion, which enlarges concomitantly with the expanding arterial wall.

Once the condensed mesenchyme begins to differentiate into cartilage, the surrounding mesenchyme starts to lose its integrity by degradation of the extracellular matrix. This is evident by focal absence of staining in the Alcian blue preparations. In Ferguson stage 20, the first signs of the developing sinus of Valsalva are apparent in the septal cushion followed by the appearance of endocardial strands invading the septal cushion from both aortic sides. We could not establish whether the endothelial strands sprout from the endothelium of the sinus or arise de novo from the mesenchyme of the septal cushion. By stage 21, the endothelial strands have met end to end to form a tunnel in the septal cushion penetrating the advancing IA. In stage 24, the tunnel through the septal cushion in caiman has reached an estimated diameter of about 5%–10% of the systemic aorta. We have to realize that the main intracardiac communication, the oval foramen between the right and the left atrium, is far greater, allowing much more blood shunting from right to left before hatching. With respect to the pre-hatching amounts of blood involved, we assume that the physiological function of the FOP is restricted and may even be negligible. During the adult cardiac cycle, the net flow through the FOP is also very small to non-existent [17] as the left ventricular pressure in the systemic aorta surmounts the pressure in the visceral aorta. The attributed functions of the FOP have been associated with many activities including diving or pH-related postprandial metabolism. The functions [19,61] also require the contraction of specialized muscle nodules (dubbed “cogteeth” or “cogwheels” [62,63]) in the right ventricular outflow to increase the right ventricular blood pressure over a threshold to allow the mechanism of opening (and closing) of the foramen by the guarding septal valve leaflet in the systemic aorta [64].

Intriguingly, the cartilage prongs discussed above are spatially associated with the FOP and the ostium of the systemic aorta, but less with that of the visceral aorta. It is tempting to propose a mechanical function in maintaining the size of both these conduits when the open FOP allows right ventricular blood into the systemic aorta.

## Figures and Tables

**Table 1:** Number of embryos and stages used. Fer Ferguson stage (crocodilians), compared with HH Hamburger and Hamilton stage (chicken), and Th Teiler stage (mouse).

Caiman	Crocodile	Alligator	Fer Stage	HH Stage	Th Stage
1	1	5	17	31	21
1	1	1	19	32	22
2		1	20	35	23
2			21/2	36	25
1			24	40	
1	1		25	40+	
		1	26		
		2	27		
			28 hatched	45 hatched	27 born

## Data Availability

All data are included in this manuscript.

## Supplementary Materials

The following are available online at https://www.mdpi.com/article/10.3390/jcdd8100132/s1, Figure S1: showing the relations in crocodilians between the cardiac chambers and the arteries, FP foramen of Panizza located between systemic (right-sided, RAo) aorta, emerging fom the left ventricle, and the vis-ceral (left-sided, LAo) aorta, emerging from the right ventricle (adapted from [9]. Cc carotid arteries, LA left atium, LV left ventricle, PA pulmonary arteries, PV pulmonary veins, RA right atrium, RV right ventricle, SC subclavian arteries, VC cardinal veins. Note the caudal connection between visceral aorta (LAo), providing the gut, and the systemic aorta (RAo).

## Abbreviations

AP: aorto-pulmonary septum; Apc: aortic parietal cushion; AV: atrioventricular cushion; ca: carotid artery; dAo: dorsal aorta; FS: folding septum; ggl ganglion; IA: interaortic septum; IS: inflow septum; LA: left atrium; LV: left ventricle; LVOT: left ventricular outflow tract; MB: main bronchi; ms: membranous septum between LV and RV; myo myocardium; Oes: esophagus; p: foramen of Panizza; Ppc pulmonary parietal cushion; Pu: pulmonary trunk; RA: right atrium; RV: right ventricle; RVOT: right ventricular outflow tract; sAo: systemic aorta; sc: septal cushion; SV: sinus of Valsalva; T: trachea; vAo: visceral aorta; # ventral myocardium, part of AP septum; * (presumably) left-sided neural crest cells, part of AP septum; + (presumably) right-sided neural crest, part of IA septum; Δ dorsal myocardium, part of AP septum.
