# Supplementary material for: Ventricular Septation and Outflow Tract Development in Crocodilians Result in Two Aortas with Bicuspid Semilunar Valves
Source: J Cardiovasc Dev Dis. 2021 Oct 15;8(10):132. doi: 10.3390/jcdd8100132 (PMC8537894; doi:10.3390/jcdd8100132)
Supplement: Supplementary file 1 [file jcdd-08-00132-s001.zip › jcdd-1340060-supplementary.pdf]

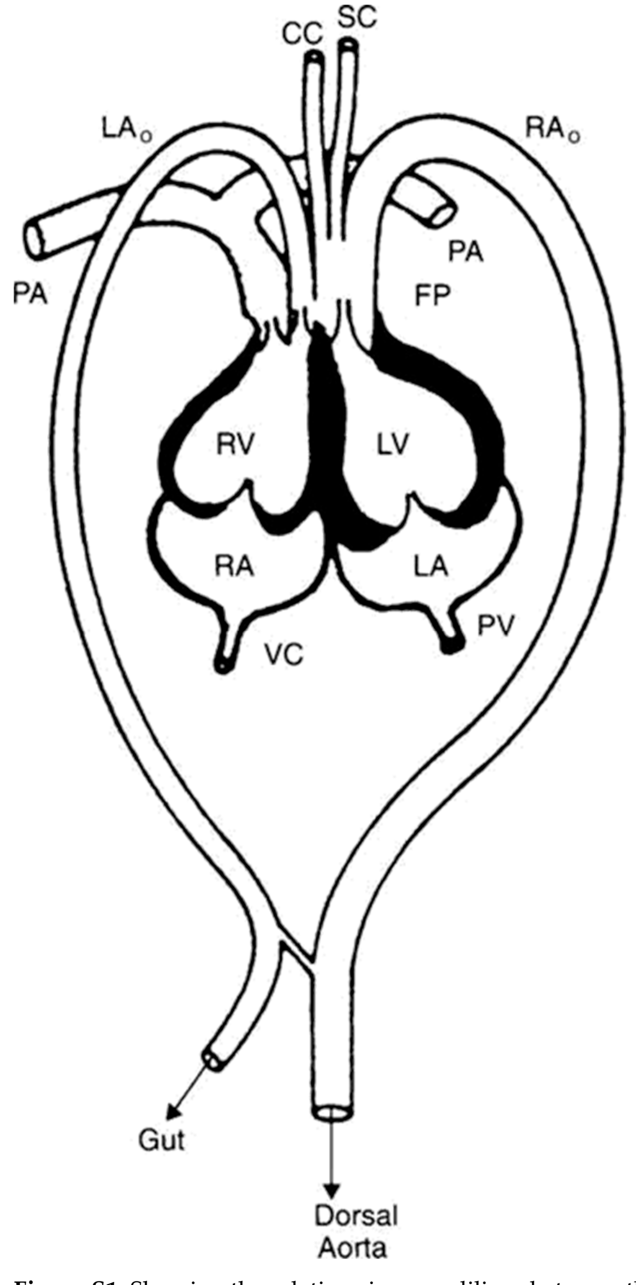

Figure S1. Showing the relations in crocodilians between the cardiac chambers and the arteries, FP foramen of Panizza located between systemic (right-sided, RAo) aorta, emerging fom the left ventricle, and the vis-ceral (left-sided, LAo) aorta, emerging from the right ventricle (adapted from [9]. Cc carotid arteries, LA left atium, LV left ventricle, PA pulmonary arteries, PV pulmonary veins, RA right atrium, RV right ventricle, SC subclavian arteries, VC cardinal veins. Note the caudal connection between visceral aorta (LAo), providing the gut, and the systemic aorta (RAo).
